# Release of Si from Silicon, a Ferrosilicon (FeSi) Alloy and a Synthetic Silicate Mineral in Simulated Biological Media

**DOI:** 10.1371/journal.pone.0107668

**Published:** 2014-09-16

**Authors:** Gunilla Herting, Tao Jiang, Carin Sjöstedt, Inger Odnevall Wallinder

**Affiliations:** KTH Royal Institute of Technology, Division of Surface and Corrosion Science, School of Chemical Science and Engineering, Stockholm, Sweden; Université de Technologie de Compiègne, France

## Abstract

Unique quantitative bioaccessibility data has been generated, and the influence of surface/material and test media characteristics on the elemental release process were assessed for silicon containing materials in specific synthetic body fluids at certain time periods at a fixed loading. The metal release test protocol, elaborated by the KTH team, has previously been used for classification, ranking, and screening of different alloys and metals. Time resolved elemental release of Si, Fe and Al from particles, sized less than 50 µm, of two grades of metallurgical silicon (high purity silicon, SiHG, low purity silicon, SiLG), an alloy (ferrosilicon, FeSi) and a mineral (aluminium silicate, AlSi) has been investigated in synthetic body fluids of varying pH, composition and complexation capacity, simple models of for example dermal contact and digestion scenarios. Individual methods for analysis of released Si (as silicic acid, Si(OH)_4_) in synthetic body fluids using GF-AAS were developed for each fluid including optimisation of solution pH and graphite furnace parameters. The release of Si from the two metallurgical silicon grades was strongly dependent on both pH and media composition with the highest release in pH neutral media. No similar effect was observed for the FeSi alloy or the aluminium silicate mineral. Surface adsorption of phosphate and lactic acid were believed to hinder the release of Si whereas the presence of citric acid enhanced the release as a result of surface complexation. An increased presence of Al and Fe in the material (low purity metalloid, alloy or mineral) resulted in a reduced release of Si in pH neutral media. The release of Si was enhanced for all materials with Al at their outermost surface in acetic media.

## Introduction

Silicon is the second most abundant element found in the Earths crust and is commonly found as silica (SiO_2_) or as silicates, the latter being the most abundant mineral group [1], [2].

The silicon surface is, unless modified, covered with a few nm thick layer of amorphous silica [3], [4]. Such an amorphous layer causes an otherwise crystalline material to behave similarly to amorphous silica in terms of dissolution [5]. As a metalloid, silicon possess desirable properties applicable for semiconductors and solar cells, but it is also widely used in glasses, ceramics and refractory materials and as an alloying element for many steel grades and aluminium alloys. This may cause human exposures to a wide variety of silicon containing powder particles at *e.g.* occupational settings. A vast number of investigations on the stability of engineered silica particles in humans and their dissolution properties have been performed during the past 60 years [4]. Airborne particles are associated with adverse effects on human health [4] and several studies using both in-vitro and in-vivo approaches have been performed to evaluate how humans may be affected by for example silica and asbestos [6]–[11]. These studies have shown silica dissolution to depend on pH [11]–[13], particle size [14]–[16], presence of cations and salts [17]–[26] and presence of complexing agents [27]–[30]. However, only few studies have addressed silica dissolution in synthetic biological solutions [4], [11].

The dissolution process of silica in water is depolymerisation through hydrolysis, where the hydroxyl ion, OH^−^, acts as a catalyst and temporarily changes the coordination number of silicon atoms on the surface resulting in weaker oxygen bonds to the underlying bulk silicon [4]. In alkaline solutions the initiation of the process is governed by adsorption of the hydroxyl ion followed by release of Si(OH)_5^−^_ into solution [4]. Below pH 11 silicon is quickly hydrolysed to Si(OH)_4_ and OH^−^, and the hydroxyl ion is free to repeat the process. This process is depressed at acidic conditions. Some hydroxyl ions are also involved in the formation of Si(OH)_5^−^_. Above pH 11 is Si(OH)_4_ converted to Si(OH)_5^−^_ resulting in a non-saturated solution and a continued dissolution of silica [4].

Aluminium has been shown to reduce the solubility of silica at alkaline conditions through different mechanisms. At a weakly alkaline pH, Al in solution forms negatively charged aluminosilicate sites on the silicon surface that repel OH^−^ interactions, thereby decreasing the dissolution rate of Si [19]. Even small amounts of Al, 40–100 µg/L, present in solution have proved to significantly reduce the solubility of Si [4]. Hydrolysed aluminium has at acidic conditions been shown to weaken the Si-O bonds in silica gel, thus increasing the dissolution of silicon [20].

The mechanism of Fe in combination with silica has not been as thoroughly investigated as for Al, but has shown a similar, albeit weaker, effect on the dissolution rate [4]. Other cations such as Mg^2+^, Ca^2+^, Ba^2+^, Na^+^, K^+^ and Li^+^ also have a strong effect on the dissolution of silica particles, however addition of these ions to pH neutral solutions increase dissolution of quartz [23], [24], [31], [32]. Other compounds that commonly are in contact with silica are organic acids, for example citric acid [27]–[30]. Citric acid enhances both dissolution of silicon and aluminium in the entire pH range although its effect decreases with decreasing pH [28].

A test protocol for evaluation of metal release from metals and alloys in synthetic body fluids, elaborated by the KTH team, previously used for classification, ranking and screening of different alloys and metals [33]–[35] was utilised to obtain data relevant for risk assessment within the scope of REACH. The aims of this investigation were to fill knowledge gaps related to the release of silicon and other elements from particles of silicon metalloids of different purity, a ferrosilicon alloy and an aluminium silicate mineral in different synthetic biological fluids, and to investigate whether the release behaviour could be related to the bulk and/or surface composition of the materials. The synthetic fluids are simple models for different human exposure routes of particles of relevance for inhalation, dermal contact and ingestion [36]–[39].

## Materials and Methods

### Materials

Four different silicon containing materials, two grades of metallurgical silicon (SiHG, SiLG), one ferrosilicon alloy (FeSi) and one aluminium silicate mineral, (Mullite, AlSi) were investigated, table 1. As three of the test items (SiHG, SiLG and FeSi) are commercially relevant in different solid forms and particle sizes, they were pre-treated (crushed, sieved, re-crushed), still maintaining their general material characteristics, to generate particles sized less than 50 µm of relevance to human inhalation, dermal contact and digestion. Crushing was accomplished using a Retsch Jaw crusher with crushing jaws made of manganese steel and wearing plates of stainless steel. Generated items less than 5 mm in size were separated from larger items and consecutively sieved until the desired size, smaller than 50 µm, was obtained. The entire procedure was performed at the Norwegian University of Science and Technology. The aluminium silicate, AlSi, powder was purchased from Sigma Aldrich.

**Table 1:** Nominal bulk composition of the investigated silicon-containing materials.

Materials	Abbreviation	Si [wt%]	Fe [wt%]	Al [wt%]	Other elements
High purity silicon	SiHG	99.1	0.4	0.1	Ca, Mn, Ti
Low purity silicon	SiLG	98.6	0.5	0.3	Ca, Mn, Ti
Ferrosilicon alloy	FeSi	75	24	0.7	Ca, C
Aluminium silicate (Mullite)	AlSi	13.2	-	38	O

Particle size distribution measurements were performed in phosphate buffered saline (PBS) using a Malvern Mastersizer 2000 laser diffraction equipment and refractive indexes for Si (3.5) and water (1.33). The specific surface area (m^2^/g) was determined by BET analysis using a Micromeritics Gemini V surface area analyser performed at Kanthal AB, Sweden. The procedure is described in detail elsewhere [40]. The specific surface area (BET) and particle size distribution of the four materials investigated in this study are given in table 2. Since SiHG, SiLG and FeSi were pre-treated in the same way, they revealed similar size distributions in PBS with a median particle size (d_0.5_) of 29.5±3.6 µm, table 2. The similarity in particle size distribution was also reflected in the specific surface areas for SiHG and SiLG (0.96 and 0.97 m^2^/g, respectively), whereas FeSi revealed a slightly smaller specific surface area (0.52 m^2^/g).

**Table 2:** Specific surface area, BET, (m^2^/g) of the different silicon-containing test items with corresponding median particle diameters (d_0.5_) and the 10% (d_0.1_) and 90% (d_0.9_) size distribution cut-off points as a percentage of volume (mass), determined using laser diffraction.

Test item	d_0.1_ [µm]	d_0.5_ [µm]	d_0.9_ [µm]	BET- surface area [m^2^/g]
SiHG	6.7	29.4	70.9	0.96
SiLG	5.8	26.0	67.8	0.97
FeSi	8.3	33.1	75.6	0.52
AlSi	16.0	30.2	53.1	0.19

Particle shape and morphology were investigated by means of scanning electron microscopy (SEM), with a Hitachi TM100 instrument. All images were obtained in back-scattered electron mode with an acceleration voltage of 15 keV.

**Figure 1:**
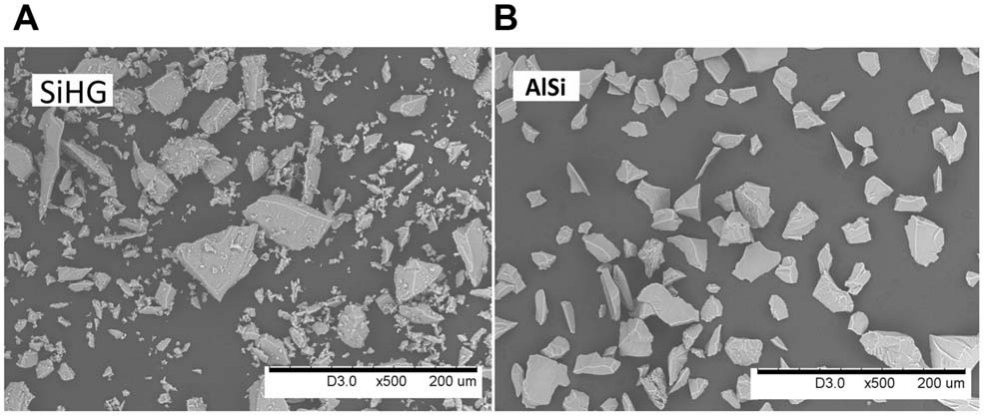
SEM backscatter images of representative particle morphologies of Si HG, SiLG and FeSi, illustrated by Si HG (A) and the AlSi mineral (B).

The commercially relevant materials (Si HG, Si LG, FeSi) displayed a similar visual appearance, illustrated by SiHG, Fig. 1, (A). Sharp edged particles of varying size ranging from larger (50–100 µm) to smaller sizes (<10 µm) were evident. The smaller sized particles were predominantly present at the surface of the larger sized particles. The AlSi mineral, Fig. 1 (B), revealed the most uniform size distribution with only few particles smaller than 10 µm.

X-ray photoelectron spectroscopy (XPS) was employed to evaluate the chemical composition of the outermost surface layer (5–10 nm) using an UltraDLD spectrometer from Kratos Analytical, Manchester, UK, with a monochromatic Al-Kα x-ray source (10 mA, 15 kV). Wide spectra and detailed high resolution spectra of Si 2p, Al 2p, Fe 2p, O 1s and C 1s were run. All binding energies were calibrated by assigning the carbon-hydrocarbon peak (C–H, or C–C) to 285 eV. All peak areas were determined by assigning a linear base line.

### Elemental release

To avoid any risk of contamination all experiments were performed using acid-cleaned lab equipment, 10% HNO_3_ for at least 24 h, followed by rinsing four times with ultra-pure water (MilliQ, 18.2 MΩcm) and drying in ambient laboratory air. As a precaution against contamination of Si no glassware was used in the experimental work. Release studies were carried out using triplicate samples of each material exposed for 2, 4, 8, 24 and 168 h. In addition, one blank reference sample for each material and time period containing only the test solution was incubated together with the triplicates. Four different synthetic body fluids were investigated, phosphate buffered saline (PBS), artificial sweat (ASW), artificial lysosomal fluid (ALF) and artificial gastric fluid (GST). Their chemical compositions and pH are presented in table 3.

**Table 3:** Chemical composition (g/L) and pH of the different synthetic biological fluids [56]–[58].

Chemicals	PBS	ASW	ALF	GST
MgCl_2_			0.050	
NaCl	8.77	5	3.21	
Na_2_HPO_4_	1.28		0.071	
Na_2_SO_4_			0.039	
CaCl_2_•2H_2_O			0.128	
C_6_H_5_Na_3_O_7_•2H_2_O (sodium citrate)			0.077	
NaOH			6.00	
Citric acid			20.8	
Glycine			0.059	
C_4_H_4_O_6_Na_2_•2H_2_O (Na_2_Tartrate•2H_2_O)			0.090	
C_3_H_5_NaO_3_ (NaLactate)			0.085	
C_3_H_5_O_3_Na (NaPyruvate)			0.086	
KH_2_PO_4_	1.36			
Urea		1		
Lactic acid		940 µL		
HCl				10
**pH**	**7.2**		**4.5**	**1.7**

5±0.5 mg of each material was weighed in a TPX Nalge jar using a Mettler AT20 balance with readability of 2 µg. 50 mL of the test solution was then added to the TPX Nalge jar containing the powder sample before being incubated in a Stuart platform-rocker incubator, regulated at 37±0.5°C. The solutions were gently shaken (bi-linearly) at an intensity of 25 cycles per minute.

After the test periods, the samples were allowed to reach ambient room temperature before the final pH of the test solution was measured. The test media was then separated from the powder particles by centrifugation at 3000 rpm for 10 min resulting in a visually clear supernatant with remaining particles in the bottom of the centrifuging tube. The supernatant was carefully poured into 25 mL HDPE bottles for storage before analysis. An efficient removal of all particles from the supernatant was confirmed by dynamic light scattering (Malvern Zetasizer nano ZS instrument).

Graphite furnace analysis GF-AAS (Perkin–Elmer AAnalyst 800 instrument) was conducted to determine total released concentrations of Al, Si and Fe, without considering their chemical speciation. Therefore the released elements are denoted only as Al, Si and Fe. All parameters used for analysis are presented in table 4.

**Table 4:** AAS analytical parameters for each element.

	Pre-treatment	Furnace program			Wavelength	Injection volume	Matrix modifier	Calibration standards
Element		T [°C]	Ramptime [s]	Holdtime [s]	[nm]	[µL]		[µg/L]
Si	pH adjustment to pH 8–9	110	1	30	251.6	15	Mg(NO_3_)_2_ Ca(NO_3_)_2_	0, 600, 1000
	pH adjustment to pH 8–9	130	20	35	251.6	15	Mg(NO_3_)_2_ Ca(NO_3_)_2_	0, 600, 1000
	pH adjustment to pH 8–9	1200	10	20	251.6	15	Mg(NO_3_)_2_ Ca(NO_3_)_2_	0, 600, 1000
	pH adjustment to pH 8–9	2350	0	5	251.6	15	Mg(NO_3_)_2_ Ca(NO_3_)_2_	0, 600, 1000
	pH adjustment to pH 8–9	2600	1	5	251.6	15	Mg(NO_3_)_2_ Ca(NO_3_)_2_	0, 600, 1000
Fe	pH adjustment to pH <2	110	1	35	248.3	20	Mg(NO_3_)_2_	0, 50, 100, 300
	pH adjustment to pH <2	130	20	30	248.3	20	Mg(NO_3_)_2_	0, 50, 100, 300
	pH adjustment to pH <2	1400	10	20	248.3	20	Mg(NO_3_)_2_	0, 50, 100, 300
	pH adjustment to pH <2	2100	0	5	248.3	20	Mg(NO_3_)_2_	0, 50, 100, 300
	pH adjustment to pH <2	2550	1	3	248.3	20	Mg(NO_3_)_2_	0, 50, 100, 300
Al	pH adjustment to pH <2	110	10	40	309.3	15	Mg(NO_3_)_2_ Pd(NO_3_)_2_	0, 30, 60, 100
	pH adjustment to pH <2	130	15	40	309.3	15	Mg(NO_3_)_2_ Pd(NO_3_)_2_	0, 30, 60, 100
	pH adjustment to pH <2	1200	10	20	309.3	15	Mg(NO_3_)_2_ Pd(NO_3_)_2_	0, 30, 60, 100
	pH adjustment to pH <2	2400	0	5	309.3	15	Mg(NO_3_)_2_ Pd(NO_3_)_2_	0, 30, 60, 100
	pH adjustment to pH <2	2450	1	3	309.3	15	Mg(NO_3_)_2_ Pd(NO_3_)_2_	0, 30, 60, 100

The instrument was calibrated prior to each set of samples to be analysed and the calibration curves were repeatedly verified during analysis by running quality control samples of known concentration every 6 to 8 samples. Mean released concentrations were based on five (Si) or three (Al and Fe) replicate readings of each sample. The limits of detection for total Al, Si and Fe in the various test media are presented in table 5.

**Table 5:** Limits of detection for total Si, Al and Fe in different test media by means of AAS-GF analysis [µg/L] (Si and Al) and AAS-F.

Element	PBS [µg/L]	ASW [µg/L]	ALF [µg/L]	GST [µg/L]
Si	45	10	15	5
Al	1.0	1.0	0.5	2.0
Fe	12	15	20	12

The resulting supernatant was poured into 25 mL HDPE bottles. Samples destined for Al and Fe analysis were acidified (pH <2) with 65% supra pure HNO_3_. In contrast to most elements, like Fe and Al, which require acidic solutions for total metal concentration analysis, analysis of total Si is best performed at alkaline conditions (pH 8–8.5). Samples exposed in PBS were analysed without pH adjustment whereas samples exposed to the other test fluid (ASW, ALF and GST) were adjusted to pH 8–8.5 using NaOH (ASW- 1% NaOH; ALF-25% NaOH; GST 8% NaOH). The concentration of NaOH used for each fluid was determined by the buffering effect of each fluid as as addition of as small amounts of NaOH as possible was desirable to minimise dilution effects. pH was measured before and after adjustment to ensure the desired effect.

At acidic conditions, silica precipitates and form compounds and complexes that lead to an underestimation of its total concentration. As a consequence, neutral or weakly alkaline solutions are essential to analyse total released Si since these conditions break the bonds of silicon complexes [41]. For reproducible analysis of total Si in the four different test fluids, each fluid had to be treated individually and operational methods for the graphite furnace elaborated for each fluid and element. This involved for example prolonged time steps and raised temperatures compared with recommended standard values.

A universal method of improving metal analysis with GF-AAS is to use matrix modifiers [42]. For Si it is recommended to use Mg(NO_3_)_2_, or sometimes Ca(NO_3_)_2_, to improve the reproducibility. During the method development it was concluded that a combination of the two modifiers in a solution of pH between 8 and 9 generated the best results, c.f. Table 5.

Another reported possibility, other than having weakly alkaline conditions to improve the reproducibility of the Si analysis is to add hydrogen fluoride, HF. However, tests performed concluded negligible improvement of the reproducibility compared with the current set-up.

**Figure 2:**
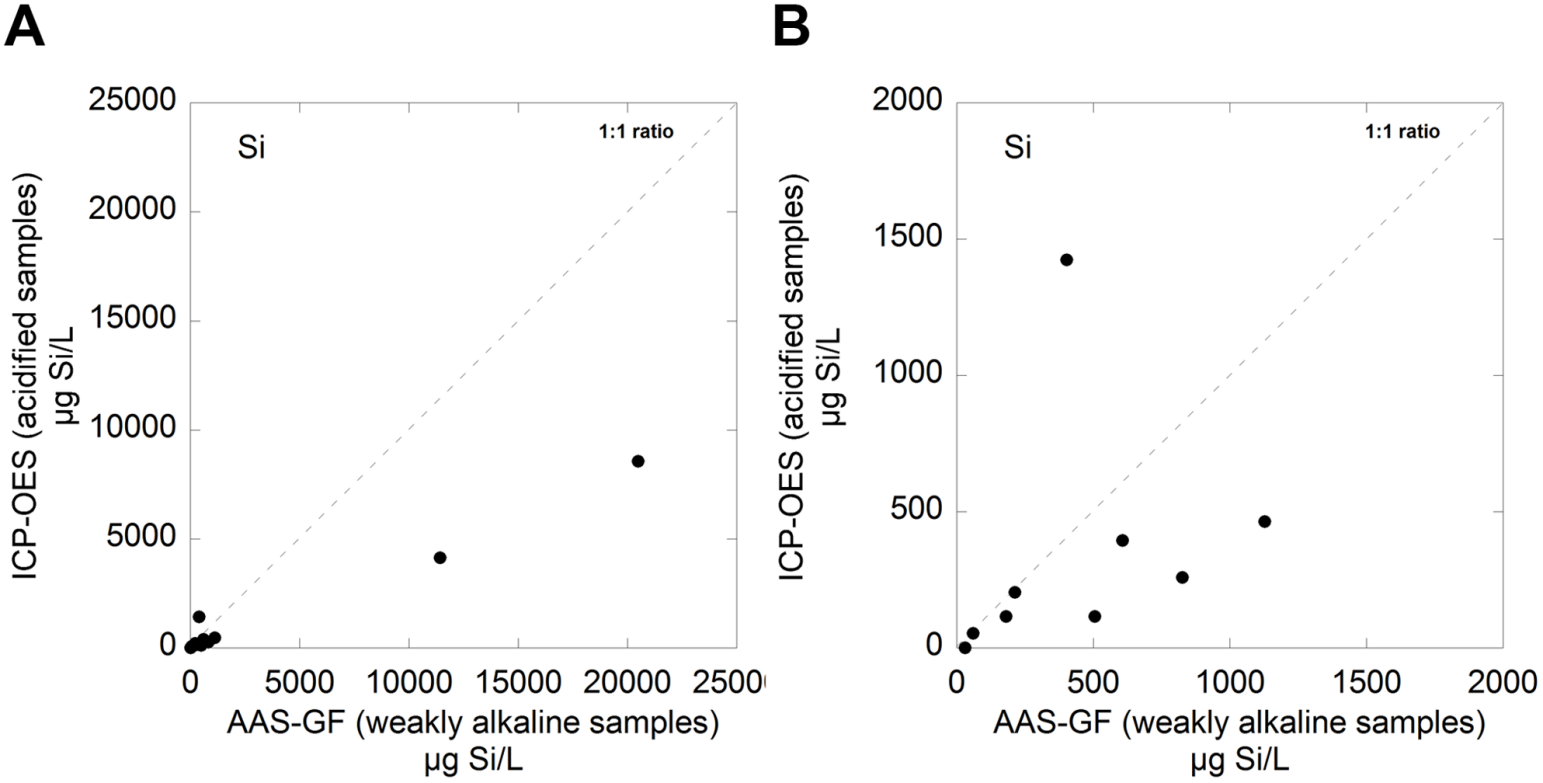
Silicon analysis by means of ICP-OES (acidified samples) compared with AAS-GF (weakly alkaline samples). A magnification of the (A) figure is given in (B).

Released concentrations of silicon measured with the elaborated GF-AAS protocol were compared determined with inductively coupled plasma – optical emission spectroscopy, ICP-OES, standard measurements (acidified samples). The results are presented Figure 2 and clearly illustrate that ICP analysis on acidic samples significantly underestimates the released silicon concentration (at both low and high released concentrations).

The recommended furnace programs for Al and Fe were used with a minimum of modification of experimental parameters.

### Chemical speciation modeling

The results from exposures in PBS and GST were used to perform chemical equilibrium speciation modeling to evaluate possible precipitation of complexes and minerals. The Visual MINTEQ software code version 3.0 [43] was used for the calculations with all relevant equilibrium constants based on the NIST compilation [44] included. Concentrations of Cl^−^, Na^+^, K^+^, PO_4_^3-^ in the test media were used as input data in the model together with the total Si concentrations measured in the supernatants by AAS entered as H_4_SiO_4_ (aq). Determined concentrations of Al and Fe were entered as trivalent cations. The pH values measured at the end of the experiments were included and the temperature was set to 37°C. The equilibrium speciation was calculated and minerals with saturation indices exceeding 0 were recorded. The minerals considered, their solubility constants and reaction enthalpies are presented in Table 6.

**Table 6:** Si-, Al-, and Fe-containing minerals and their solubility constants (K_s_) and enthalpies of reaction as included in the Visual MINTEQ database.

Mineral	Chemical formula	Log *K*_s_	ΔH_r_ [kJ/mol]
Chalcedony	SiO_2_	−3.55	19.70
Cristobalite	SiO_2_	−3.35	20.01
Quartz	SiO_2_	−4.00	22.36
SiO_2_ (am, gel)	SiO_2_	−2.71	14.00
SiO_2_ (am, ppt)	SiO_2_	−2.74	15.15
Al(OH)_3_ (am)	Al(OH)_3_	10.80	−111.00
Al(OH)_3_ (Soil)	Al(OH)_3_	8.29	−105.00
Al_2_O_3_(s)	Al_2_O_3_(s)	19.65	−258.59
Boehmite	γ-AlO(OH)	8.58	−117.70
Diaspore	α-AlO(OH)	6.87	−103.05
Gibbsite (C)	Al(OH)_3_	7.74	−105.00
Variscite	AlPO_4_·2 H_2_O	−22.07	−9.40
Halloysite	Al_2_Si_2_O_5_(OH)_4_	9.57	−181.43
Imogolite	Al_2_SiO_3_(OH)_4_	13.00	−193.60
Kaolinite	Al_2_Si_2_O_5_(OH)_4_	7.44	−148.00
Fe(OH)_2.7_Cl_0.3_ (s)	Fe(OH)_2.7_Cl_0.3_	−3.04	0.00
Ferrihydrite	Fe(OH)_3_	3.20	−100.40
Ferrihydrite (aged)	Fe(OH)_3_	2.69	−100.40
Goethite	α-FeO(OH)	0.49	−60.58
Hematite	α-Fe_2_O_3_	−1.42	−128.99
Lepidocrocite	γ-FeO(OH)	1.37	0.00
Maghemite	γ-Fe2O3	6.39	0.00
Strengite	FePO_4_·2 H_2_O	−26.40	−9.36

## Results and Discussion

**Figure 3:**
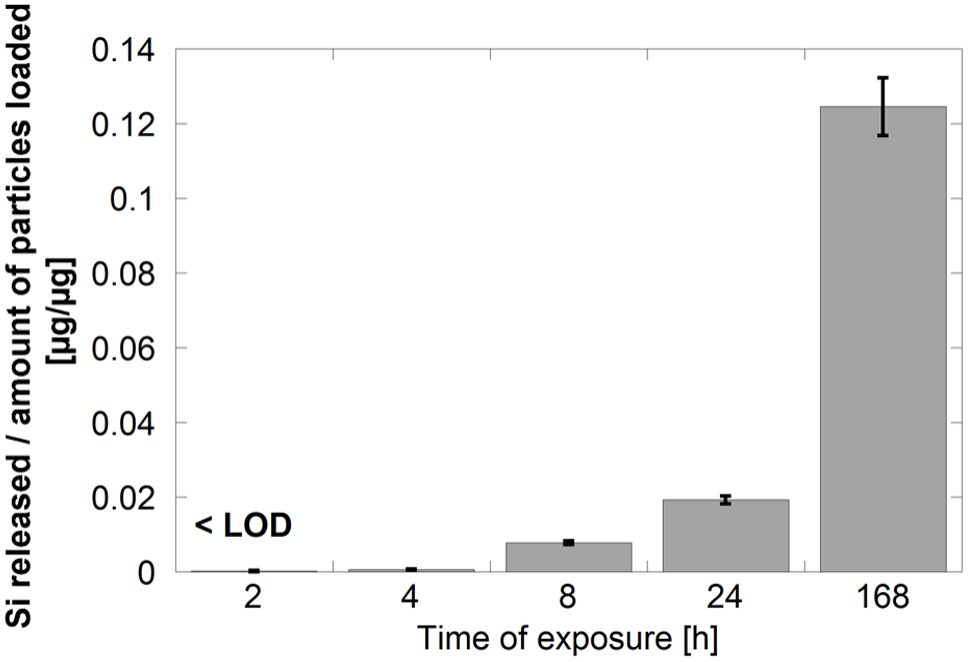
Time-dependent release of Si from SIHG in PBS (pH 7.2) per amount of loaded particles.

All released metal species are in the following denoted as the total released amount of metal/element, as Si, Al, and Fe, without considering their individual speciation. As will be discussed below the conduct of release of Si from the silicon-based materials differs with material, time, pH and fluid composition. Release data after 168 h of exposure will in the following be used to illustrate these effects as the short-time periods in many cases reveal released amounts of Si close to, or below the limit of detection for the given fluid, exemplified for SiHG in PBS, Figure 3.

### Proton- and complexation-induced release of Si from metalloidal silicon

**Figure 4:**
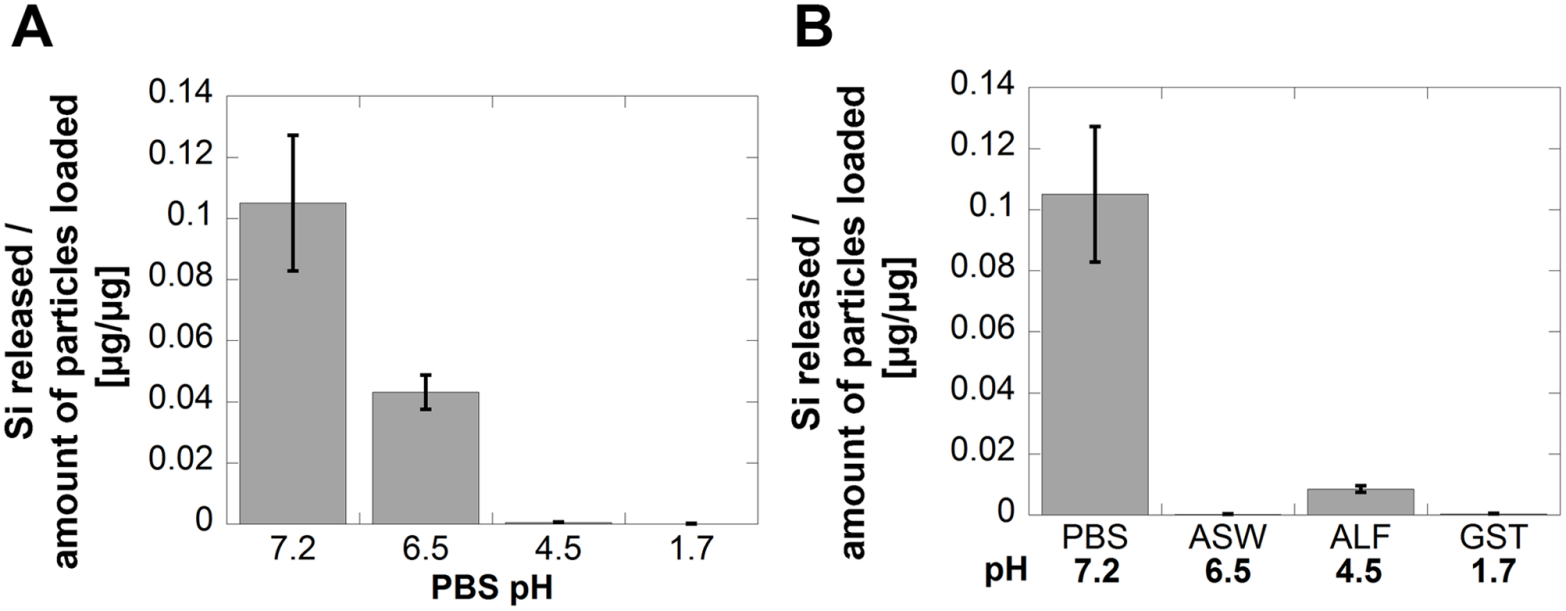
Released fractions [µg Si/µg] of Si from SiHG immersed in PBS of pH 7.2 and pH-adjusted PBS solutions (A), and corresponding released fractions of Si after 168 h of exposure in synthetic biological fluids of similar pH (GST, ALF and ASW) (B).

To assess the influence of test media pH on the amount of released Si (as silicic acid), Si HG (high grade metalloidal silicon) was exposed in PBS (pH 7.2) for 168 h and compared with parallel measurements in pH adjusted PBS solutions (pH 6.5, 4.5 and 1.7). These pH levels were selected based on acidity (not compositional differences) of artificial sweat (ASW), artificial lysosomal fluid (ALF) and gastric juice (GST). A pH effect was evident with significantly more Si released from SiHG at near-neutral pH conditions compared with PBS of lower pH, Fig. 4 (A). The measured amount of Si released from SiHG after 168 h in PBS of pH 7.2 was in the same range as literature findings in Ringer’s solution [4], however, comparisons with previously reported findings must cautiously approached as several experimental parameters such as particle loading, particle size and exposure times are different. The release of Si exponentially decreased with reduced pH, Figure 4 (A). These results are in agreement with data reported in literature as the solubility of Si is well known to be higher in pH neutral and alkaline solutions compared with acidic solutions [1], [12]. The substantial reduction of released Si between pH 6.5 and 4.5 (a factor of 85), Fig. 3 (A), was supported by literature findings [45] that showed the dissolution of Si to be proportional to the hydroxyl ion concentration in the pH interval between 3 and 6. Above pH 6 the rate limiting step for dissolution is the desorption rate of Si(OH)_5^−^_ from the surface [4].

**Figure 5:**
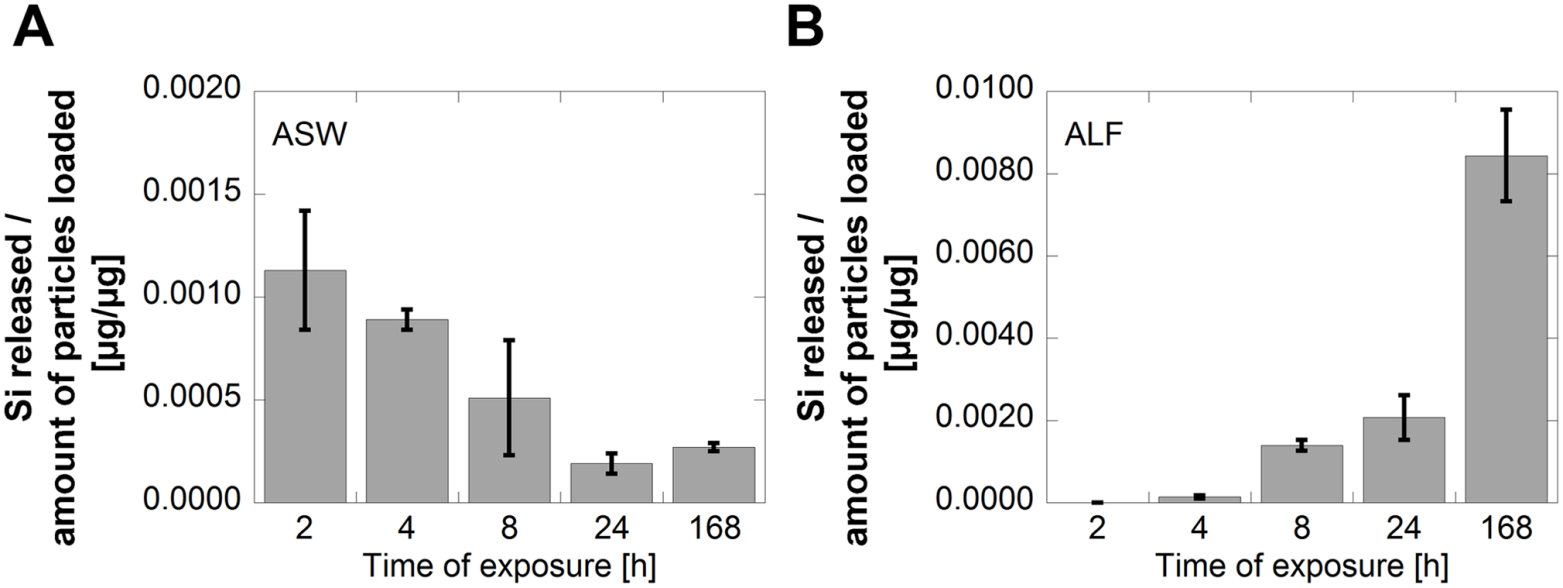
Time-dependent released amounts of Si per amount of loaded particles of SiHG in ASW, pH 6.5 (A), and ALF, pH 4.5 (B).

When comparing the release of Si from Si HG in the pH adjusted PBS solutions with parallel exposures in synthetic body fluids of corresponding pH (but different composition), the same general trend with higher release of Si at pH neutral conditions compared with lower pH conditions was observed. However, the results clearly demonstrate the additional importance of fluid constituents. This is particularly evident when comparing the release behaviour of Si in ASW (pH 6.5) and the pH- adjusted PBS to the same pH (pH 6.5), Fig. 4. The measured amount of Si in solution after 168 h in ASW was only a fraction of the corresponding release in PBS. This either suggests adsorption of for example urea at the SiHG surface through hydrogen bonding [46] with silanol surface groups that hinders the hydroxyl-induced release of Si, or that released Si form large complexes with fluid components (urea, lactic acid and chlorides) that sediment or precipitate with time, and are therefore not measured in solution. These effects are evident from the kinetic investigation in ASW with reduced levels of released Si in solution with time, Figure 5, (A). Both explanations are plausible but cannot be distinguished from each other. Exposures in ALF on the other hand showed the opposite situation, with enhanced Si levels in solution (almost 15 times) in ALF compared with PBS of the same pH, Figure 4. Here, the solution components seem to enhance the dissolution of Si with time as illustrated with time-dependent findings, Figure 5 (B).

The most plausible explanation for enhanced release of Si from SiHG in ALF is related to the adsorption of ligands (citrate) that form weak organic complexes with silanol groups at the surface and with silicic acid in solution. Ligand-adsorption on metal oxides in solution and its importance for the metal release/dissolution mechanisms have previously been reported in literature [47]–[50]. The effect is for example documented for stainless steel particles releasing ions of Cr, Fe, Mn and Ni [51] where the release of chromium ions, known to have a strong complexation ability with citric acid, increased non-linearly with increasing citric acid concentration. This effect, though less pronounced, has been shown for silicon [27] where a concentration of 6 g/L citric acid was reported to yield a 10% increase in silicon solubility at neutral pH values. This is in line with the observed release of Si in ALF (20.8 g/L citric acid) where the released amount did not present a 3.5 fold increase compared with literature findings [27]. However, ALF is a more complex solution containing several other components that also may influence the silicon release process compared with the pure citric acid solution.

**Figure 6:**
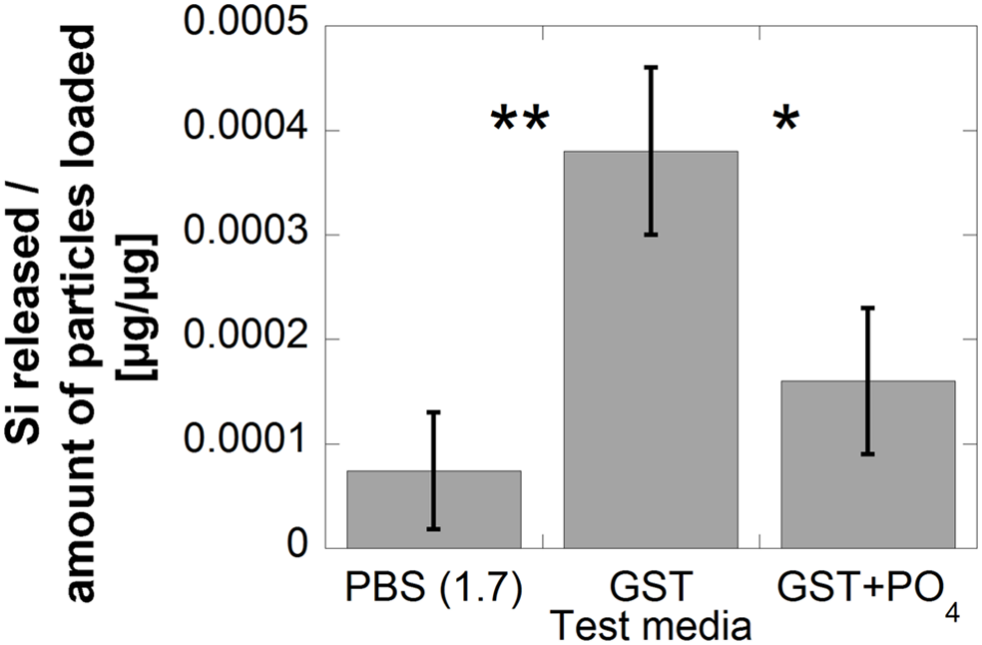
Released fractions of Si [µgSi/µg] from SiHG immersed in PBS (pH 1.7), GST and GST+PO_4_^2-^ (phosphate concentration corresponding to PBS) after 168 h of exposure at 37°C. Error bars represent the standard deviation of triplicate samples, except for PBS (pH 7.2) where six samples were investigated. The asterisks refer to significance levels (Student’s t-test) for the test media compared with each other; p<0.05 (*) and p<0.01 (**).

Low released amounts of Si were observed from SiHG in both PBS and GST of pH 1.7, Figure 4. It was, however, evident that even at these low levels the release of Si in GST were still significantly higher (p<0.01) compared with PBS (pH 1.7), Figure 6. As GST consists of ultra-pure water and analytical grade HCl it does not contain any components that should act as complexing agents thereby promoting the release process. Literature findings claim that chlorides have no observed effect on silica solubility [25], [26]. Phosphate species in PBS may interact and adsorb on the surface and thereby hinder the release of Si at these conditions. Similar observations have previously been observed for example for stainless steel in pH-neutral lung-simulated fluids [33]. Phosphate added to the GST fluid in concentrations similar to PBS, significantly reduced the release of Si (p<0.05), Fig. 6. Literature findings show that hydrogen bonds can form between phosphates and non-ionised silanol surface groups in fluids of low pH (<pK_a_ of surface silanol groups) [52]. These bonds reduce the number of silanol groups available for interactions with hydroxyl ions, thereby reducing the total silicon release. Alkali cations such as Na^+^ have been reported to promote dissolution of silica [17], [22], [23]. As this effect not could be observed for PBS at pH 1.7 (containing 8.8 g NaCl/L) when compared with GST (no Na) it is likely that the hindering effect of surface-linked phosphates is stronger than the accelerating effect of Na.

### Release of Si from metalloidal silicon compared with a ferrosilicon alloy and a silicate mineral

**Figure 7:**
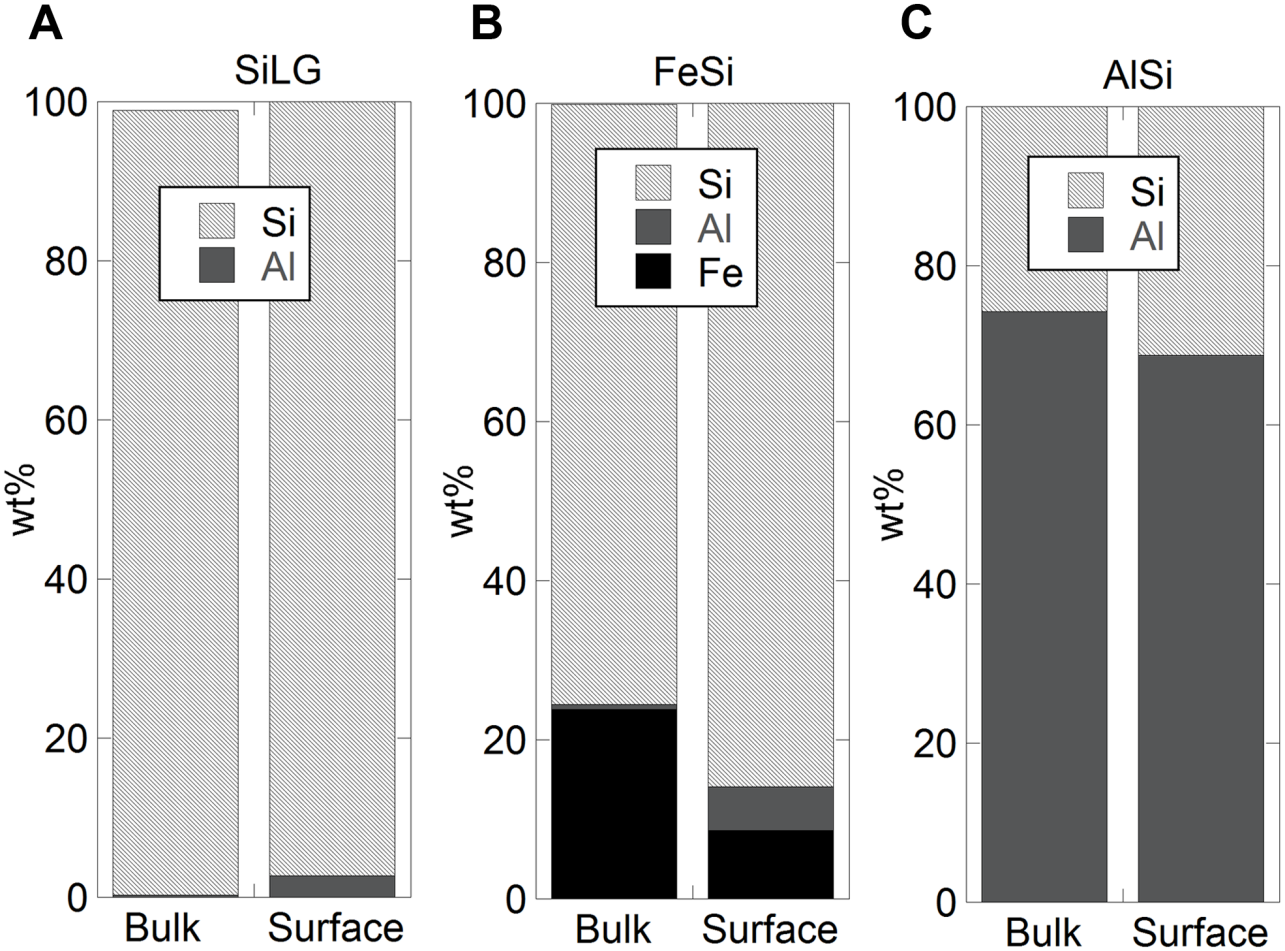
Relative surface composition of oxidised Si, Al and Fe of a silicon metalloid, SiLG (A), an alloy, FeSi (B) and a mineral, AlSi (C), determined by means of XPS, compared with the nominal bulk composition based on supplier information.

Availability of data in literature on the release of silicon and other metals from sparingly soluble inorganic silicon metalloid substances of different purity, compounds and alloys in synthetic biological fluids is scarce and its magnitude cannot be predicted based the nominal bulk composition. The presence of impurities and alloying elements will alter the material properties, the outermost surface configuration and inevitably the metal release process. Differences in bulk and surface composition between a silicon metalloid, SiLG, an alloy, FeSi, and a mineral, AlSi are presented in Figure 7.

Both the metalloids, SiHG and SiLG, and the ferrosilicon alloy, FeSi, revealed Si 2p peaks at 99.2±0.2 eV (2p_3/2_) and 99.7±0.4 eV (2p_1/2_), characteristic of non-oxidised silicon, and broad peaks centred at 103.0±0.2 eV, assigned as four-valent silicon. The latter peak is consistent with literature findings for silica, SiO_2_ (Teixeira et al, 2005; Sun et al, 2000; Hansch et al, 1999), an assignment supported by an oxygen (O1s) peak at 532.4±0.1 eV (Wagner et al, 1982). The oxygen peak can also be assigned to oxidised carbon and for SiLG to oxidised aluminium. Low quantities of other SiO_x_ suboxides such as Si_2_O_3_, SiO, Si_2_O, or polysubstitutional tetrahedral Si-(O_4-n_Si_n_) (n = 0–4) are likely due to peak widths slightly exceeding literature findings for SiO_2_ (Teixeira et al, 2005). The observation of peaks assigned to non-oxidised silicon indicate thin nanometer-thick surface oxides predominantly composed of silica (SiO_2_).

Small amounts of oxidised aluminium (Al 2p at 74.5x±0.2 eV) were observed on the surface for SiLG. The same peak was present both for the FeSi alloy and the AlSi mineral in increasing relative amounts, Figure 6. Oxidised iron (Fe 2p centred at 706.7±0.2 eV, dominating, and 710.5±0.5 eV) were present at the FeSi surface. The proportion of Si, Al and Fe in the outermost surface layer was not proportional to the bulk alloy composition, Figure 7.

**Figure 8:**
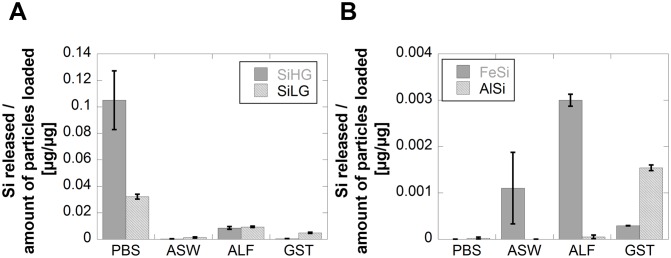
Comparison of released amounts of Si per amount of loaded particles from SiHG and SiLG in PBS, ASW, ALF and GST (A), and from FeSi and AlSi in the same test media (B) after 168 h of exposure.

Differences in released amounts of Si are presented in Figure 8 for the metalloids, the alloy and the mineral after 168 h of exposure in the different synthetic biological fluids. Despite similar particle sizes and surface areas for SiHG and SiLG, table 2, the release of Si from SiHG was 4 times higher compared with SiLG in the pH neutral media PBS. Non-significant, or opposite findings (5 and 2 times higher released amount of Si from Si LG compared with Si HG in ASW and GST, respectively), were observed in fluids of high complexation capacity or low pH. The results suggest that even small amounts of oxidised aluminium as found on the surface of SiLG, Figure 7, influence the release mechanism of Si. Such an effect could not be predicted using the nominal bulk composition only as a measure of the released proportion of elements. The results are in agreement with literature findings showing Al in solution, or on the surface, to hinder the dissolution process of Si in weakly alkaline solutions [4], [17], [19], [20], [29], [53].

Oxidised aluminium on the SiLG surface most likely weakens surface-silanol bondings at acidic conditions thereby enhancing the release of Si from SiLG compared with SiHG. The only exception was in ALF where similar amounts of Si were released from SiHG and SiLG. However, in ALF the citrate-induced dissolution effect is, as previously discussed, believed to dominate.

Significantly lower amounts of Si were released from the alloy and the mineral compared with the silicon metalloids of different purity in the synthetic biological fluids, Figure 8 (B). The previously observed pH effect for the silicon metalloids was not evident for these materials despite a large relative fraction of oxidised silicon on the outermost surface for FeSi (85%) and AlSi (30%). No measurable amounts of released Si in solution were evident for the silicon-, iron-, and aluminium- rich surface oxide of the FeSi alloy in PBS after 168 h. Such a mixed oxide film has previously been shown to be more stable compared with surface oxides of the each component individually [4]. This mixed oxide was less stable in the acidic GST fluid in which the amount of released Si was similar to observations for SiHG despite a lower relative surface content of oxidised silicon. Most Si, although at low levels, was released in ASW and ALF, probably triggered by released Al, see below, and complexing agents, *c.f.* previous discussion.

Approximately 12 wt% of the total silicon content of the SiHG particles were released after 168 h in PBS, which is almost four times more than released from the low grade metalloid, SiLG (3.2 wt%) with a surface oxide predominantly consisting of silicon-oxides and small (5%) amounts of Al as oxides. Only 0.02% of the amount of silicon in the loaded particles of AlSi, with almost 30 wt% oxidised aluminium in its outermost surface, Figure 7, was released in PBS after 168 h. Non-significant amounts of Si released from the FeSi alloy were measured in solution.

**Figure 9:**
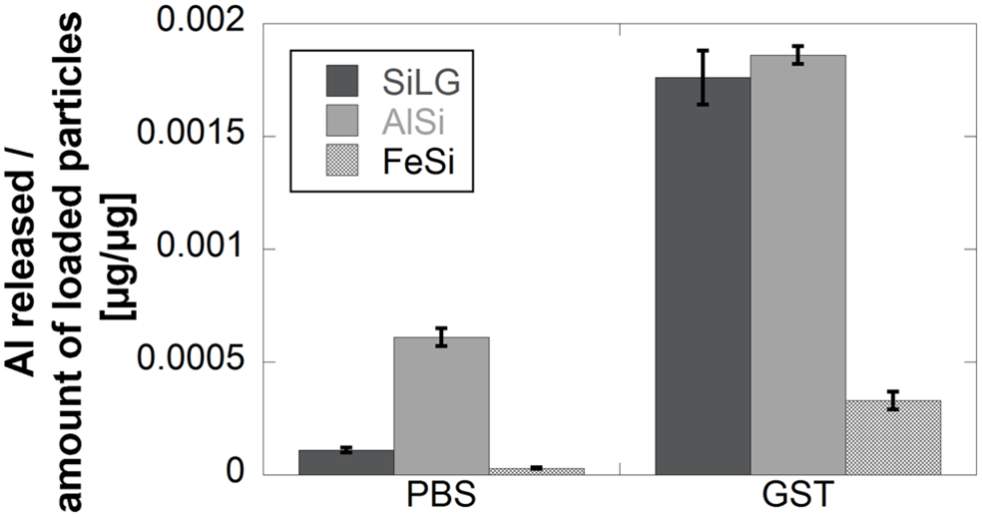
Comparison of released amounts of Al per amount of loaded particles of SiLG, AlSi and FeSi exposed in PBS (pH 7.2) and GST (pH 1.7) for 168 h.

Released amounts of Si from the AlSi mineral were below (in ASW) or close to the limits of detection (in PBS and ALF) except for the most acidic medium (GST), in which the release was significantly higher, albeit still low. These findings are opposite to observations for the silicon metalloids that revealed most Si released in near-neutral pH fluids. The discrepancy is believed to be attributed the release of aluminium from the AlSi-mineral. The release of Al was generally more pronounced in the acidic media of GST compared with the pH neutral PBS, Figure 9.

A relative concentration of 5% oxidised aluminium was present in the outermost surface layer even though the bulk content of Al in SiLG was as a low as 0.3 wt%, table 1. The release of Al into solution after exposure for 168 h in GST was equivalent to 62% of the aluminium bulk content. Significantly lower amounts of Al (0.5 wt%) were released from aluminium silicate in GST after 168 h when compared with its bulk content. More Si was released from AlSi in GST compared with PBS. Corresponding fractions in PBS of near-neutral conditions were 3.9 and 4.9% for SiLG and AlSi, respectively.

**Figure 10:**
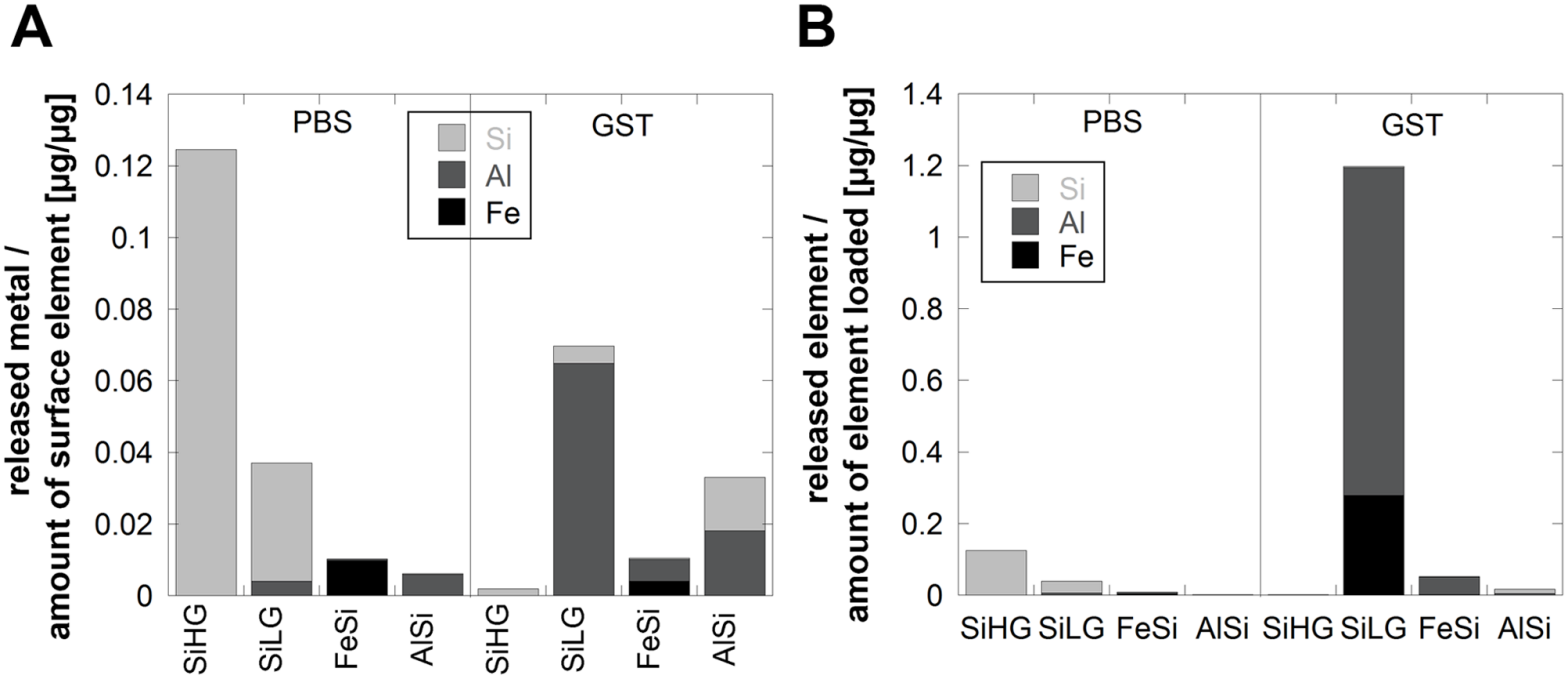
Comparison of released amounts of Si, Al and Fe per amount of each element in the surface oxide (A) and in the bulk (B) of SiHG, SiLG, FeSi and AlSi, exposed in PBS (pH 7.2), and GST (pH 1.7) for 168 h.

The released amounts of Al from the FeSi alloy compared with its bulk content were approximately 5 wt% in GST and 0.4 wt% in PBS. Less than 0.3% of the amount of loaded particles was released as iron, Fig. 10.

No correlation was evident between the released elemental proportion, Figure 10, and their bulk or surface composition, Figure 7 (XPS).

### Chemical speciation modelling

Chemical equilibrium speciation modelling, using the Visual MINTEQ 3.0 software [43], was performed for the GST and PBS test media to predict chemical speciation of released species and identify potential precipitation of various minerals. The possible interaction of Al released from the surface and present in solution, was also estimated. Quartz was the only mineral that could precipitate in PBS at given released concentrations of Si from SiHG. However, according to literature findings the kinetics at 37°C are very slow and any precipitation would take centuries [54]. This suggests that measured concentrations of Si in solution actually reflect its total release as no precipitation of Si-containing compounds is predicted to take place within the 168 h experiment. Similar conclusions were drawn for released Si from SiLG, FeSi and AlSi of lower concentrations.

The additional release of Al (from SiLG, FeSi and AlSi) in different concentrations may to some extent precipitate as Al(OH)_3_ or AlPO_4_·2 H_2_O in both PBS and GST, and to a small extent as Al_2_Si_2_O_5_(OH)_4_. Measured released concentrations of Al in solution may therefore be slightly underestimated. Released Fe may result in the formation and precipitation of ferrihydrite Fe(OH)_3_, goethite α-FeO(OH), hematite α-Fe_2_O_3_, lepidocrocite γ-FeO(OH), maghemite γ-Fe_2_O_3_, and Fe(OH)_2.7_Cl_0.3_. However, saturation indices above 0 indicate that released iron in solution was present as small aqueous colloids of these phases, not possible to remove by centrifugation, rather than free ions. Measured released iron concentrations are therefore reflecting total released quantities.

The model further predicts that Fe(OH)_2.7_Cl_0.3_ may precipitate in GST. However, its presence is of low probability [55], and if formed, it would be present as small colloids in solution.

Any precipitation effect in ASW and ALF could not be modelled because of their more complex compositions.

## Conclusions

Elemental release studies have been performed on two grades of metallurgical silicon (high purity silicon, SiHG, low purity silicon, SiLG), a ferrosilicon alloy (FeSi) and an aluminium silicate mineral (Mullite, AlSi) in a selection of biological test fluids of varying pH, composition and complexation capacity aiming to generate unique quantitative bioaccessibility data and to assess the influence of surface/material and test media characteristics on the elemental release process.

Main observations are summarised below:

- The silicon grades of different purity revealed a strong pH dependence with the highest release of Si at near-neutral conditions triggered by hydroxyl-silanol interactions, an effect that logarithmically decreased with reduced pH to minor levels.
- The complexation capacity of the biological fluids strongly influenced the release of Si. In artificial sweat, ASW pH 6.5, the measured released amount of Si in solution decreased with time. This effect was attributed to the adsorption of for example urea and lactic acid on the SiHG surface through hydrogen bonding [46] with silanol surface groups. As a consequence the hydroxyl-induced release of Si was hindered, or the released Si formed large complexes with fluid components (urea, lactic acid and chlorides) that setteled or precipitated with time, and were therefore not measured in solution.
- The measured amount of released Si in solution from SiHG exposed in artificial lysosomal fluid, ALF (pH 4.5), of strong complexation capacity, increased with time, and considerable more Si was released from SiHG in ALF, compared with the PBS pH-adjusted to 4.5. This was attributed to the formation of weak organic surface complexes between citrate and silanol groups at the surface and with silicic acid in solution. Similar ligand-induced dissolution processes have been reported for metals and metal oxides.
- The proportion of Si, Al and Fe in the outermost surface layer of the low purity silicon, the ferrosilicon alloy and the aluminium silicate mineral was not proportional to their bulk compositions. Silicon was present as silica, most likely amorphous, in the outermost layer of SiHG and SiLG. Silicon is present as an oxide of silica, aluminium- and iron oxides on FeSi. Si is present as Si(IV) both in the bulk and at the surface for AlSi (Mullite), as expected.
- The presence of oxidised Al on the outermost surface strongly reduced the release of Si in pH neutral media. Even small amounts (3 wt%) of oxidised Al for the low-grade silicon, SiLG, reduced the release of Si by a factor 4 in PBS (pH 7.2) compared with SiHG with no oxidised Al at the surface. The Si-, Al- and Fe-rich oxide on FeSi was stable at pH-neutral conditions reducing the release of Si to levels close to the limit of detection. However, its stability was reduced at acidic conditions releasing Fe and Al as a result. The aluminium silicate, Mullite, AlSi was poorly soluble at pH-neutral conditions, an effect that was somewhat reduced at acidic conditions.
- Based on chemical speciation modelling using the Visual MINTEQ software released Si in solution did not precipitate in PBS or GST, whereas some precipitation may occur for Al as Al(OH)_3_ or AlPO_4_·2 H_2_O. Measured released concentrations of Al in solution may therefore be slightly underestimated, whereas measured Si concentrations in solution reflect the total amount of released Si. Released Fe and Al were, in addition, predicted to form small colloids of oxides and hydroxides consequently staying in solution. Precipitation effects in ALF and ASW were not possible to predict with the model.
- Specific operational methods for silicon analysis with GF-AAS were elaborated and protocols established for each test fluid to enable measurements of reliable data on total silicon concentrations in solution. This involved optimisation of graphite furnace step temperatures and times, the use of a combination of Ca(NO_3_)_2_ and Mg(NO_3_)_2_ as matrix modifiers and pH solution adjustment to 8–9. These protocols and GF-AAS analyses were proven more accurate than ICP-OES measurements performed on acidified solutions following standard operational procedures.
